# Numerical Analysis of Bifacial Photovoltaic Panels Subjected to Repeated Loading

**DOI:** 10.3390/ma19081630

**Published:** 2026-04-18

**Authors:** Tomasz Jankowiak, Jan Białasik, Magdalena Łasecka-Plura, Mieczysław Kuczma

**Affiliations:** 1 Institute of Structural Analysis, Poznan University of Technology, Piotrowo 5, 60-965 Poznań, Poland; tomasz.jankowiak@put.poznan.pl (T.J.); magdalena.lasecka-plura@put.poznan.pl (M.Ł.-P.); 2 Institute of Building Engineering, Poznan University of Technology, Piotrowo 5, 60-965 Poznań, Poland; jan.bialasik@put.poznan.pl

**Keywords:** bifacial photovoltaic module, finite element analysis, repeated loading, cell spacing, laminated glass, mechanical reliability, composite action

## Abstract

This study investigates the mechanical response of bifacial glass–glass photovoltaic modules subjected to snow-type loading, with a particular focus on the influence of silicon cell spacing on global deformation and local stress distributions in the silicon cell layer and the adhesive layer. Five computational finite element models were developed which explicitly represent all laminate layers and discrete cell layout. The numerical results are interpreted within the framework of partial interaction and shear transfer between the glass plies and are validated against previously obtained home conducted experimental observations. The results demonstrate that a silicon cell layout has a pronounced effect on local tensile stresses in silicon cells and on the curvature distribution within the laminate, while its influence on the global kinematic response is less critical. The numerical analysis indicates that the relative displacements between the glass layers resulting from the flexibility of the adhesive bond play a critical role.

## 1. Introduction

Bifacial photovoltaic (PV) modules have become a dominant technology in modern utility-scale solar power plants due to their increased energy yield and potential for improved durability under realistic field conditions [1,2,3,4]. Unlike monofacial modules, bifacial panels commonly employ glass–glass laminates and quasi-symmetric layer configurations, which not only increase flexural stiffness but also enable rear-side energy harvesting. However, their mechanical response is governed not only by the global geometry of the laminate but also by the internal architecture of the silicon cell layout, including the spacing between cells and the resulting discontinuities in the stiff silicon layer.

Bifacial PV panels are typically composed of rigid front and rear glass layers, with solar cells encapsulated within a compliant polymer interlayer, such as polyolefin elastomer (POE) or ethylene-vinyl acetate (EVA), which transfers shear between glass layers and governs composite action [5,6,7]. In real installations, PV modules are subjected to repeated or quasi-cyclic loading due to snow accumulation, wind gusts, and temperature variations [8,9]. Such actions can induce microcracking in brittle silicon cells and may lead to gradual power degradation, especially when local stress concentrations develop at cell edges, interconnects, and layer interfaces [10,11]. Despite numerous experimental and numerical studies on the mechanical performance of PV modules under static loading, the influence of cell spacing and discrete cell layouts on the global stiffness and local stress fields of bifacial glass–glass laminates remains insufficiently explored, particularly in configurations motivated by building-integrated PV (BIPV), where cell discontinuities are intentionally introduced to increase transparency or enable architectural patterns [12].

The contemporary development of BIPV technologies includes not only classical silicon-based solutions, but also advanced semi-transparent thin-film technologies, in particular perovskite solar cells, which are considered especially for windows, facades, and other energy-active glazing applications. In this context, not only their energy-related performance but also their impact on indoor visual comfort is being investigated [13]. Semi-transparent perovskite solar cells are regarded as a promising solution for BIPV due to their favorable photoelectric properties, bandgap tunability, flexibility, and potential for application in energy-generating windows and facades [14]. Experimental studies have also shown that high-performance semi-transparent perovskite solar cells with bidirectional transparent electrodes can achieve parameters indicating their practical applicability, for example in smart windows [15]. Other features that are particularly important from the viewpoint of architectural applications include transparency, color tunability, response under diffuse light, and suitability for vertical surfaces [16]. The development of semi-transparent perovskite solar cells covers a broad range of issues related to fabrication, structural engineering, applications, bifacial configurations, and implementation challenges [17]. At the same time, more detailed material and layered solutions are being developed to improve their optical and electrical properties, thereby broadening the technological background of research on modern PV module architectures [18].

From a numerical modeling perspective, photovoltaic modules are most commonly represented using homogenized shell or equivalent plate formulations [12,19]. Such approaches are computationally efficient and have been widely adopted for global stiffness assessment and impact-related analyses, including simulations of hailstone loading on PV modules [20]. In these models, the multilayer glass-cell laminate is typically reduced to an effective section with smeared material properties, enabling efficient prediction of global deformation and stress levels. However, the discrete nature of the silicon cells and the locally varying interaction between the glass plies are not represented explicitly, which limits the ability of shell-based models to capture global stiffness changes arising from non-uniform cell layouts and extended regions of reduced interlayer shear transfer [21].

An alternative modeling strategy relies on fully three-dimensional solid representations, in which the laminate layers and the silicon cells are modeled explicitly [11]. Such approaches have been employed to resolve local stress states in the silicon layer and to analyse mechanically induced damage mechanisms, including stress concentrations associated with cell geometry and material heterogeneity [11,22]. While these models provide valuable insight into local mechanical phenomena, their high computational cost and modeling complexity restrict their applicability in systematic parametric studies at the panel scale. To alleviate this limitation, hybrid modeling strategies combining a global simplified representation with locally refined three-dimensional submodels have been proposed, allowing detailed stress evaluation in critical regions while maintaining acceptable computational efficiency [12,23]. Despite these advances, systematic comparisons between homogenized shell-based models and fully three-dimensional approaches for bifacial glass–glass photovoltaic modules remain limited, particularly with respect to the role of interlayer modeling assumptions and their influence on global deflection under realistic snow-type loading conditions and non-uniform cell spacing.

This motivates numerical analyses capable of decoupling the effects of cell spacing from interlayer shear transfer. In a previous experimental study by the authors, full-scale bifacial modules with different cell spacings were subjected to repeated mechanical loading representative of snow and wind actions [24]. That study indicated a measurable reduction in global stiffness associated with increasing cell gaps and revealed pronounced deflections and residual deformations under sustained loading, while no measurable degradation of electrical performance was observed within the tested regime. These observations suggest that the governing mechanism is related to a loss of effective composite action rather than to material damage.

In contrast to existing studies, which typically rely either on homogenized shell formulations or detailed local three-dimensional models, the present work provides a systematic comparison of modeling strategies with different levels of interlayer representation. The novelty lies in the combined analysis of discrete cell layouts, interlayer shear transfer mechanisms, and boundary conditions, and in identifying global kinematic constraints as the dominant factor controlling stiffness reduction in panels with sparse cell distributions.

The objective of this study is threefold: (i) to quantify the influence of cell spacing on global stiffness through detailed 3D finite element modeling, (ii) to validate interlayer shear transfer assumptions against experimental deflections, and (iii) to establish guidelines for efficient modeling of sparse-cell PV configurations. To this end, a detailed three-dimensional finite element model is developed and used to systematically assess different interlayer modeling strategies. The numerical results are interpreted within the framework of partial interaction and shear transfer between the glass plies, and are validated against previously obtained experimental observations.

## 2. Description of the Photovoltaic Module and Loading Protocol

### 2.1. Geometry and Layer Configuration

**Figure 1:**
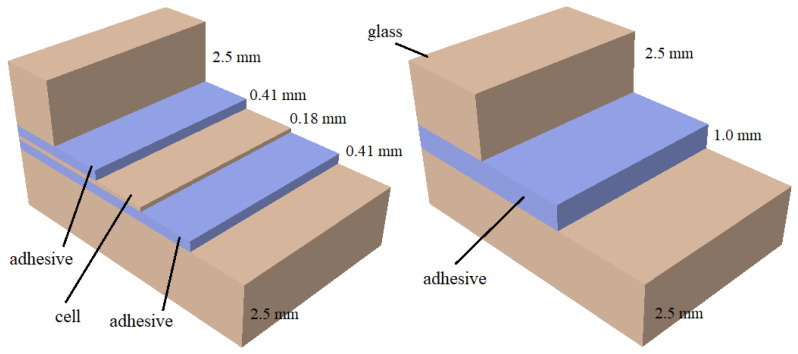
Compositions of the PV panels (cell region—**left**, between cells—**right**).

The analysed bifacial module has nominal in-plane dimensions of 1664 mm × 996 mm and a total laminate thickness of 6.0 mm. It consists of five functional layers, see Figure 1: front tempered glass, POE encapsulant, monocrystalline HJT (heterojunction technology) silicon cells, rear POE, and back glass. The thicknesses of individual layers are:

- front glass: 2.5 mm,
- front POE: 0.41 mm,
- silicon cells: 0.18 mm,
- rear POE: 0.41 mm,
- rear glass: 2.5 mm.

Monocrystalline HJT cells have in-plane dimensions of 156.75×156.75 mm. In the regions between the photovoltaic cells, the laminate consists of three layers (right in Figure 1), namely two glass plies with a thickness of 2.5 mm each and an adhesive interlayer with a thickness of 1 mm, resulting in a total thickness of 6 mm.

The layer stack is symmetric with respect to the mid-plane of the laminate, which is relevant for the homogenized shell model.

### 2.2. Cell Layout Configurations

Three silicon cell layouts are considered in order to investigate the effect of cell spacing (cell gap) on the mechanical response:

- Group I: 60 cells with a narrow spacing of approximately 2 mm,
- Group II: 40 cells with a spacing of 40 mm,
- Group III: 40 cells with a spacing of 80 mm.

These configurations represent realistic design variations in modern bifacial modules and significantly modify the effective active area and local bending stiffness of the laminate.

### 2.3. Mounting Conditions and Loading Protocol

**Figure 2:**
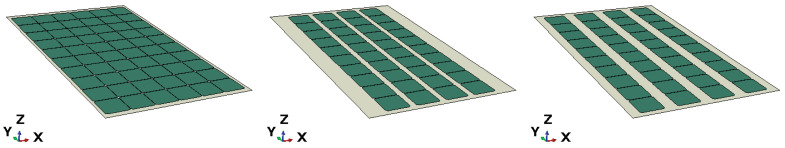
Distribution of the silicone cells in photovoltaic panels.

For each of the module groups defined above, three specimens were tested in the laboratory [24]. The modules were supported on three line supports along each of the two longitudinal edges in the Y-direction, using standard brackets and clamps in accordance with the manufacturer’s installation guidelines. Each support had an effective contact length of 108 mm, and the spacing between adjacent supports was 611 mm. The supports restrained out-of-plane displacement $u_{z}$, while in-plane translation in the X-direction ($u_{x}$) was released to allow longitudinal sliding of the module (Figure 2). This arrangement reproduces realistic mounting conditions and prevents artificial membrane stiffening.

A uniform pressure of 2.4 kPa was applied using small sandbags weighing 5 kg each, placed normal to the front glass surface to simulate a characteristic snow load. The load was maintained for one hour to allow deformation stabilization in accordance with IEC 61215-1:2021 [8]. During the tests, displacements were observed to stabilize after approximately 30 min. Although experimental campaigns may involve multiple loading cycles, the present numerical analysis considers first a single representative loading event, followed by a six-cycle sign-changing loading–unloading program, as the structural response is predominantly elastic.

Vertical displacements of the panel, $u_{z}$, were measured using three LVDT sensors installed beneath the panel: one at mid-span and two others located in the same cross-section at one-quarter and three-quarters of the span along the shorter side (X-direction). The instantaneous deflections at the central point were typically 14–17% greater than those at the corresponding left and right points across all groups. The left-right asymmetry was small (≤1.1 mm, <3%), while the differences between measurements on the top and bottom surfaces (after reversing the panel) at the central point did not exceed 1.1 mm (<3%), confirming the largely symmetric response of the panels under uniformly distributed loading.

## 3. Material Constitutive Behaviour

Based on the authors’ previous experimental investigations of full-scale bifacial panels [24], each component material of the PV panel has been modeled using linear elastic constitutive laws [25], which are valid approximations for the panel’s response under a one-hour load action. Our experimental observations indicate that the module deflection is predominantly instantaneous and reversible; however, for a loading duration of 120 h, residual displacements were observed after unloading. These time-dependent effects are not considered herein. Accordingly, a linear elastic model is adopted in the present study as a valid approximation for short-term loading, as confirmed by the reversible response observed in both experiments and numerical simulations. Nevertheless, the residual displacements observed after prolonged loading indicate the presence of time-dependent effects, such as creep or relaxation. These effects may result from the viscoelastic behaviour of the adhesive interlayer (POE/EVA), particularly under sustained loading and varying temperature conditions, and may influence interlayer shear transfer and long-term stiffness. Geometric nonlinearity was taken into account by adopting a large-displacement kinematic formulation (NLGEOM = YES), which allows for large displacements and rotations while assuming small strains. Consequently, second-order geometric effects are captured without invoking finite-strain constitutive formulations or nonlinearity of the material.

For three-dimensional continua, the constitutive relationship is expressed in Voigt notation as

(1) σ=Dε,

where

(2) $$\sigma ={\left[\begin{array}{cccccc} \sigma_{xx} & \sigma_{yy} & \sigma_{zz} & \tau_{yz} & \tau_{xz} & \tau_{xy} \end{array}\right]}^{T},\;\varepsilon ={\left[\begin{array}{cccccc} \varepsilon_{xx} & \varepsilon_{yy} & \varepsilon_{zz} & \gamma_{yz} & \gamma_{xz} & \gamma_{xy} \end{array}\right]}^{T}.$$

The glass plies and the photovoltaic cells were modeled as isotropic linear elastic materials. For isotropic elasticity, the stiffness matrix D takes the form:

(3) $$D_{\mathrm{iso}}=\frac{E}{\left(1+\nu )(1-2\nu \right)}\left[\begin{array}{cccccc} 1-\nu & \nu & \nu & 0 & 0 & 0\\ \nu & 1-\nu & \nu & 0 & 0 & 0\\ \nu & \nu & 1-\nu & 0 & 0 & 0\\ 0 & 0 & 0 & \frac{1-2\nu }{2} & 0 & 0\\ 0 & 0 & 0 & 0 & \frac{1-2\nu }{2} & 0\\ 0 & 0 & 0 & 0 & 0 & \frac{1-2\nu }{2} \end{array}\right].$$

The glass plies were assigned a Young’s modulus of $E_{g}$ = 73 GPa and a Poisson’s ratio of $\nu_{g}=0.24$. The photovoltaic cells, made of monocrystalline silicon and embedded within the adhesive layer, were modeled with a Young’s modulus of $E_{pv}$ = 160 GPa and a Poisson’s ratio of $\nu_{pv}=0.22$. The photovoltaic cells were assumed to remain fully elastic throughout the loading range, consistent with the experimental observations.

**Figure 3:**
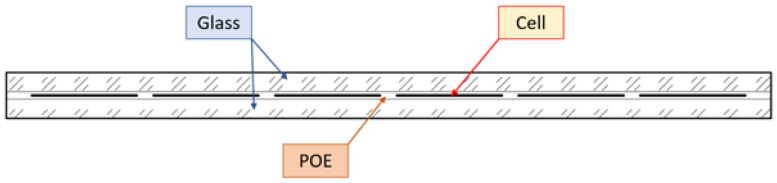
Schematic distribution of layers thought the thickness.

The adhesive interlayer was modeled using different linear elastic representations depending on the adopted modeling, explained in details in next Section 5. In continuous interlayer models, the adhesive was described as a nearly incompressible isotropic material with a Poisson’s ratio of $\nu_{a}=0.48$. The Young’s modulus of the adhesive was defined as $E_{a}$ = 10 MPa in regions beneath the photovoltaic cells and reduced to $E_{a}$ = 3 MPa in regions between the cells in order to represent spatial variations in stiffness (see Figure 1, Figure 2 and Figure 3).

In order to explicitly control shear transfer between the glass plies, selected models employed an orthotropic linear elastic formulation for the adhesive between the photovoltaic cells. In this formulation, the normal stiffness components were derived from isotropic elastic properties, while the shear moduli were defined independently. The constitutive relation is written in Voigt notation as

(4) $$\sigma =D_{\mathrm{ortho}}\varepsilon ,$$

with

(5) $$D_{\mathrm{ortho}}=\left[\begin{array}{cccccc} C_{11} & C_{12} & C_{13} & 0 & 0 & 0\\ C_{12} & C_{22} & C_{23} & 0 & 0 & 0\\ C_{13} & C_{23} & C_{33} & 0 & 0 & 0\\ 0 & 0 & 0 & G_{yz} & 0 & 0\\ 0 & 0 & 0 & 0 & G_{xz} & 0\\ 0 & 0 & 0 & 0 & 0 & G_{xy} \end{array}\right].$$

The coefficients $C_{ij}$ correspond to an equivalent isotropic material with Young’s modulus $E_{a}=10$  MPa and Poisson’s ratio $\nu_{a}=0.48$, and are defined as

(6) $$C_{11}=C_{22}=C_{33}=\frac{E_{a}\left(1-\nu_{a}\right)}{\left(1+\nu_{a}\right)\left(1-2\nu_{a}\right)}=\frac{10\times 0.52}{1.48\times 0.04}=87.8\;\mathrm{MPa},$$

(7) $$C_{12}=C_{13}=C_{23}=\frac{E_{a}\nu_{a}}{\left(1+\nu_{a}\right)\left(1-2\nu_{a}\right)}=\frac{10\times 0.48}{1.48\times 0.04}=81.1\;\mathrm{MPa}.$$

The shear moduli were defined as

(8) $$G_{xy}=G_{yz}=\frac{E_{a}}{2(1+\nu_{a})}=3.38\;\mathrm{MPa},\;G_{xz}=0.1\;\mathrm{MPa}.$$

The normal elastic constants correspond to an isotropic material with E=10MPa and ν=0.48, while the shear moduli were defined as $G_{xy}=G_{yz}=3.38\;\mathrm{MPa}$ and $G_{xz}=0.1\;\mathrm{MPa}$. This selective reduction of the shear modulus in the bending plane allows for limited interlayer shear transfer while preserving normal stiffness and through-thickness continuity.

In discontinuous interlayer models, the adhesive was retained only beneath the photovoltaic cells. The regions between the cells were modeled either by contact interactions or by cohesive elements. For contact-based models, hard contact was enforced in the normal direction, while tangential behaviour was assumed frictionless. In cohesive models, a thin cohesive layer was introduced between the glass plies in regions without adhesive. A purely elastic traction–separation law was adopted:

(9) Kδ=t,

with the stiffness matrix

(10) $$K=\left[\begin{array}{ccc} K_{n} & 0 & 0\\ 0 & K_{s} & 0\\ 0 & 0 & K_{t} \end{array}\right].$$

In Equation (9), t denotes the traction vector acting on the cohesive interface:

(11) $$t=\left[\begin{array}{c} t_{n}\\ t_{s}\\ t_{t} \end{array}\right],$$

where $t_{n}$ is the normal traction and $t_{s}$, $t_{t}$ are the two tangential traction components acting in the local interface coordinate system. The vector δ represents the relative displacement (separation) between the opposing faces of the cohesive element:

(12) $$\delta =\left[\begin{array}{c} \delta_{n}\\ \delta_{s}\\ \delta_{t} \end{array}\right],$$

with $\delta_{n}$ denoting the normal opening displacement and $\delta_{s}$, $\delta_{t}$ the tangential sliding displacements along the interface. The stiffness parameters $K_{n}$, $K_{s}$, and $K_{t}$ define the elastic penalty stiffnesses in the normal and tangential directions, respectively.

The normal stiffness was set to $K_{n}=10\;\mathrm{MPa}$ and the tangential stiffnesses were defined as $K_{s}=K_{t}=0.5\;\mathrm{MPa}$, 0.4MPa, or 0.1MPa. The adopted stiffness parameters enable independent control of normal and tangential interaction between glass plies, allowing the modeling of partial composite action without introducing damage or irreversible effects. No damage initiation or degradation mechanisms were included, ensuring fully reversible interface behaviour.

Overall, all materials were described using linear elastic constitutive laws. The experimentally observed loss of composite action was reproduced through reduced interlayer shear stiffness, interlayer discontinuities, and kinematically admissible boundary conditions combined with geometric nonlinearity, without the need to invoke finite-strain effects, plasticity, or material damage. The material parameters used in the simulations are presented in Table 1, where five computational models considered are indicated. The models are described in the next section; they differ in the adopted strategies for adhesive layer modeling (M1–M4) and in the use of a cohesive interlayer (M5).

## 4. Finite Element Discretization and Element Formulations

The numerical models were discretized using three-dimensional solid and interface finite elements available in Abaqus/Standard, selected to ensure accurate representation of bending behaviour, near-incompressible material response, and interlayer interaction. In the numerical simulations, only one half of the panel was modeled by applying a symmetry condition at the mid-span.

The glass plies were modeled using eight-node linear brick elements with incompatible modes (enhanced strain modes), C3D8I. This element formulation enriches the standard displacement field by adding incompatible displacement modes at the element level, which improves the accuracy of bending-dominated problems and mitigates parasitic shear locking commonly observed in fully integrated linear brick elements. The displacement field within the element is expressed as:

(13) $$u\left(x\right)=\sum_{i=1}^{8}N_{i}\left(x\right)u_{i}+\sum_{j=1}^{m}{\tilde{N}}_{j}\left(x\right){\tilde{u}}_{j},$$

where $N_{i}$ are the standard shape functions associated with the nodal displacements $u_{i}$, while ${\tilde{N}}_{j}$ and ${\tilde{u}}_{j}$ denote the incompatible mode shape functions and their internal degrees of freedom. These additional modes do not introduce extra nodal degrees of freedom but enhance the element strain field, resulting in improved bending performance and more accurate stress and displacement predictions.

The adhesive layer and the photovoltaic cells were modeled using eight-node hybrid brick elements (C3D8H). This element type is specifically designed for nearly incompressible materials, for which standard displacement-based formulations suffer from volumetric locking. In the hybrid formulation, the stress field is decomposed into deviatoric and volumetric parts, and the hydrostatic pressure is introduced as an independent field variable. The constitutive relation can be written as:

(14) σ=s−pI,

where s is the deviatoric stress tensor and *p* is the hydrostatic pressure, treated as an additional unknown. The weak form of equilibrium is augmented by a pressure constraint equation enforcing volumetric compatibility:

(15) $$p=-K\;\varepsilon_{v},$$

where *K* is the bulk modulus and $\varepsilon_{v}=\mathrm{tr}\left(\varepsilon \right)$ is the volumetric strain. This mixed displacement–pressure formulation effectively eliminates volumetric locking and ensures stable and accurate solutions for materials with Poisson’s ratio approaching 0.5.

Interlayer interaction in the discontinuous adhesive models was represented using eight-node three-dimensional cohesive elements (COH3D8). These elements relate the traction vector acting on the interface to the displacement jump between the opposing faces. The constitutive behaviour is governed by a traction–separation law, Equation (9) where stiffness matrix is defined as Equation (10). In the present study, the cohesive elements were employed in a purely elastic regime without damage initiation or degradation, providing a compliant but fully reversible shear connection between the glass plies.

The combined use of C3D8I, C3D8H, and COH3D8 elements enables an accurate and numerically robust representation of bending-dominated glass panels with nearly incompressible adhesive layers and spatially varying interlayer continuity, while avoiding shear and volumetric locking effects.

## 5. Numerical Analysis of Adhesive Layer Modeling and Its Influence on Panel Deflection

This section presents a systematic numerical investigation of the influence of adhesive layer modeling on the global deflection of composite glass panels with embedded photovoltaic cells. The aim is to identify the mechanical mechanisms responsible for the experimentally observed differences in deflection between panels with varying photovoltaic cell densities and to determine the minimum modeling complexity required to reproduce this behaviour numerically.

A three-dimensional solid model was employed to explicitly represent all layers of the photovoltaic module, including the glass plies, adhesive layer, silicon cells, and cell-gap regions. By resolving the full three-dimensional geometry of the laminate, the model captures local stress concentrations, stiffness discontinuities, and deformation gradients resulting from the heterogeneous material layout. These effects are particularly pronounced in regions with large cell gaps, where the local bending stiffness is reduced and curvature amplification may occur.

The analysed structure consists of two glass plies, each 2.5 mm thick, bonded by a 1 mm thick adhesive layer, within which photovoltaic cells of 0.18 mm thickness are embedded at mid-thickness. Three geometric configurations were considered: Group I, characterised by a dense distribution of cells; Group II, with a significantly lower cell density; and Group III, containing the same number of cells as Group II but distributed more uniformly. All configurations were subjected to bending loads corresponding to the experimental tests [24]. The dominant deformation mode is bending in the xz plane, with deflection occurring in the *z* direction along the panel span (*x* direction).

### 5.1. Finite Element Model and Discretization

All simulations were performed using a full three-dimensional finite element model. The glass plies were discretized using C3D8I elements to accurately capture bending behaviour without shear locking. The adhesive layer and photovoltaic cells were modeled using C3D8H hybrid elements to represent the nearly incompressible adhesive material (Poisson’s ratio ν≈0.48) and to avoid volumetric locking.

The adhesive layer was discretized with three elements through the thickness in the regions between the photovoltaic cells, resulting in an individual element thickness of approximately 0.33 mm. In regions where photovoltaic cells are present, the adhesive was discretized with one element on each side of the cell while preserving the total adhesive thickness. The in-plane element size was 5 mm. An in-plane mesh size of 10 mm and two elements through thickness (C3D8I) were used for the glass plies.

Five modeling strategies, M1–M5, were applied that differ in interlayer representation, shear transfer assumptions, boundary conditions, and their physical interpretation (Table 2).

### 5.2. Adhesive Layer Modeling Strategies

Four modeling strategies were investigated to progressively assess the influence of adhesive stiffness, shear transfer, interlayer continuity and global kinematic constraints.

#### 5.2.1. Fully Continuous Isotropic Interlayer (M1)

In the first approach, the adhesive layer was modeled as a homogeneous isotropic linear elastic material with Young’s modulus E=10 MPa, corresponding to full composite interaction between the glass plies. The resulting deflections were 27.63 mm for Group I and 28.04 mm for Group II, showing only a marginal difference between configurations. This behaviour is inconsistent with experimental observations and indicates that the assumption of full interlayer interaction is overly restrictive.

#### 5.2.2. Two-Material Isotropic Interlayer (M2)

In the second approach, a spatially heterogeneous adhesive model was introduced. The adhesive beneath the photovoltaic cells retained a stiffness of E=10 MPa, while the adhesive between the cells was assigned a reduced modulus of E=3 MPa. This modification increased the deflection of Group II to 30.16 mm, while Group I remained nearly unchanged (28.03 mm). Although partial interaction effects were promoted, the experimentally observed loss of stiffness was still underestimated.

#### 5.2.3. Orthotropic Interlayer with Reduced Shear Stiffness (M3)

In the third approach, the adhesive between the cells was modeled as an orthotropic elastic material to explicitly control shear transfer between the glass plies. Reducing all shear moduli to G=0.1 MPa increased the deflection of Group II to 31.81 mm. When only the shear modulus in the bending plane ($G_{xz}$) was reduced to 0.1 MPa while the remaining shear moduli retained isotropic values ($G_{xy}=G_{yz}$ = 3.38 MPa), the deflection reached 31.20 mm. This comparison demonstrates that shear transfer in the bending plane governs the global response, while secondary shear mechanisms provide additional but limited stiffness.

#### 5.2.4. Discontinuous Interlayer with Contact Formulation (M4)

In the fourth approach, a discontinuous interlayer model was introduced. The adhesive layer was retained only beneath the photovoltaic cells, while the regions between the cells were replaced by a contact formulation with hard contact in the normal direction and zero friction in the tangential directions. This approach eliminates artificial long-range shear transfer while preserving through-thickness continuity. With standard boundary conditions enforcing kinematic compatibility at the supports, the resulting deflections were 28.25 mm for Group I and 32.15 mm for Group II. Although the qualitative trend was captured, the magnitude of the deflection increase remained lower than observed experimentally.

### 5.3. Extension of the Model with a Cohesive Interlayer (M5)

In the first stage, the discontinuous interlayer model was extended by introducing cohesive elements in the regions between the photovoltaic cells, while retaining the standard boundary conditions applied jointly to both glass plies. A thin cohesive layer was inserted exclusively between the cells. The formulation was purely elastic and did not include any damage initiation or degradation mechanisms. The normal stiffness was set to 10 MPa, consistent with the Young’s modulus of the adhesive material, whereas the tangential stiffness was significantly reduced in order to represent limited shear transfer.

The cohesive representation does not model cracking or debonding, but provides a compliant shear connection with fully reversible behaviour. However, replacing the contact formulation with cohesive elements did not result in a significant change in the global deflection. The difference between Group I and Group II remained small, and the predicted deflections were comparable to those obtained with the contact-based model. This indicates that the introduction of a compliant cohesive interlayer alone, without modifying the boundary conditions, is insufficient to reproduce the experimentally observed loss of composite action.

### 5.4. Influence of Boundary Conditions

**Figure 4:**
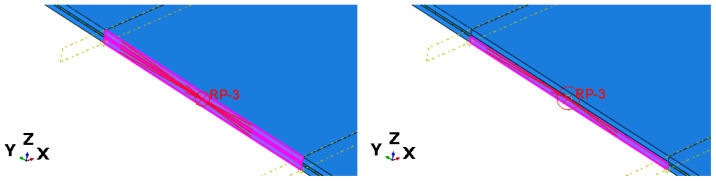
Boundary conditions with kinematic constrain to both glass layers (**left**) and one layer (**right**).

Further analysis revealed that the remaining discrepancy between numerical and experimental results originates primarily from the boundary conditions applied at the supports, Figure 4. When conventional hinged boundary conditions are imposed simultaneously on both glass plies, relative in-plane displacements are suppressed at the supports (Figure 4, left). This constraint enforces global composite action even when interlayer interaction is locally weakened or interrupted. Boundary conditions allowing independent in-plane displacements of the glass plies better represent realistic mounting conditions in photovoltaic installations. In practice, mounting systems may permit limited relative slip between layers. The numerical results demonstrate that constraining both plies jointly artificially enforces composite action and leads to underestimation of deflections. The numerical response is therefore sensitive to support stiffness, friction, and restraint conditions.

Only after applying independent boundary conditions to each glass ply, allowing relative in-plane displacements while maintaining global equilibrium, did the numerical response change significantly (Figure 4, right). In this configuration, the deflections increased to 29.24 mm for Group I, 34.88 mm for Group II, and 32.23 mm for Group III. These values are in very good agreement with the experimental measurements (the maximum relative error, observed for Group III, is 5.2%) and clearly demonstrate that the loss of composite action is governed primarily by global kinematic constraints rather than by local material damage (Table 3).

**Figure 5:**
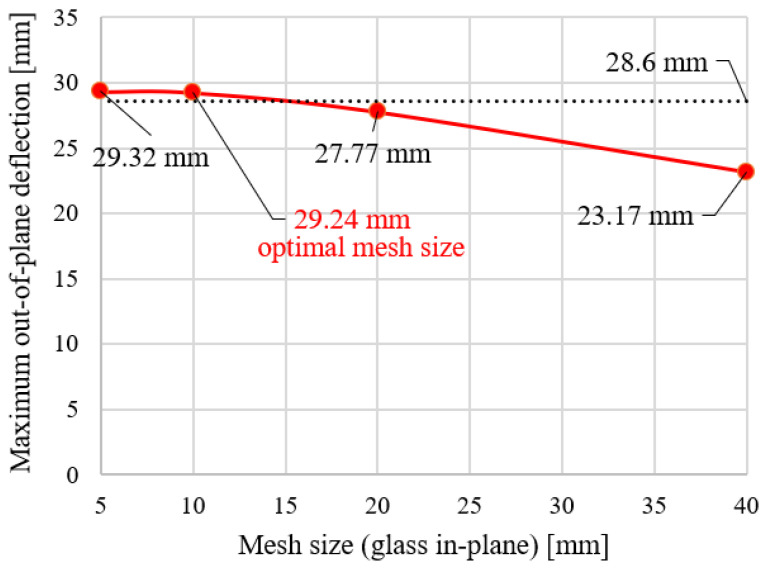
Mesh size sensitivity for group I—final model.

A mesh sensitivity study confirmed that further refinement does not significantly affect the global deflection, indicating that the observed differences are governed by modeling assumptions rather than discretization effects. All models were developed in Abaqus/Standard [25]. A mesh sensitivity study was conducted for Group I. The results are presented in Figure 5. The maximum deflection was evaluated for models discretized with element sizes of 40 mm (coarse mesh), 20 mm (medium mesh), 10 mm (fine mesh), and 5 mm (very fine mesh), as shown in Figure 5. Based on the convergence behaviour and computational efficiency, an element size of 10 mm was adopted for all groups, providing a satisfactory compromise between accuracy and computational time.

The combination of a discontinuous interlayer model with cohesive elements and kinematically admissible, independent boundary conditions therefore provides an accurate representation of both the absolute deflection levels and the differences observed between the individual panel configurations.

### 5.5. Mechanical Interpretation

The results show that the experimentally observed differences in deflection between panels with different cell densities are structural in nature. Panels with a dense distribution of photovoltaic cells (Group I) behave close to a fully composite laminate because the cells act as frequent shear bridges. In contrast, panels with sparse or uneven cell distributions (Groups II and III) contain extended regions without effective shear transfer, allowing the glass plies to deform increasingly independently.

Continuous adhesive models and boundary conditions that suppress relative displacements both artificially enforce composite action and therefore underestimate deflections in sparsely reinforced panels.

### 5.6. Theoretical Background

The observed behaviour is consistent with classical partial interaction and shear-lag theories for layered structures [26,27]. The effective bending stiffness may be expressed as

(16) $$D_{\mathrm{eff}}=EI_{\mathrm{eff}},$$

and depends primarily on the ability of the interlayer to transfer shear stresses along the span.

Within the shear-lag framework, the relative slip s(x) satisfies

(17) $$\frac{d^{2}s\left(x\right)}{dx^{2}}-\frac{k_{s}}{EI_{\mathrm{eff}}}s\left(x\right)=0,$$

leading to an interaction length

(18) $$\ell_{s}=\sqrt{\frac{EI_{\mathrm{eff}}}{k_{s}}}.$$

In photovoltaic panels, the interaction length provides a measure of the distance over which shear stresses can be effectively transferred between the glass plies. When the spacing between photovoltaic cells exceeds this characteristic length, $\ell_{s}$, composite action is progressively lost and the panel response approaches that of partially interacting layers. This mechanism operates at a global structural scale and cannot be captured by local constitutive refinements, mesh refinement or damage models.

No adhesive cracking or degradation is required to explain the experimental results. The increased deflection arises from a loss of global composite action governed by interlayer continuity and boundary conditions. A minimal discontinuous modeling strategy combined with kinematically admissible boundary conditions therefore provides the most physically justified and numerically efficient representation of the observed behaviour.

### 5.7. Distribution of Displacement and Stress in the Panels

In order to provide insight into the internal stress transfer mechanisms responsible for the differences in effective bending stiffness identified in the previous sections, the spatial distributions of displacement and stress fields obtained from the three-dimensional finite element simulations are analysed below.

**Figure 6:**
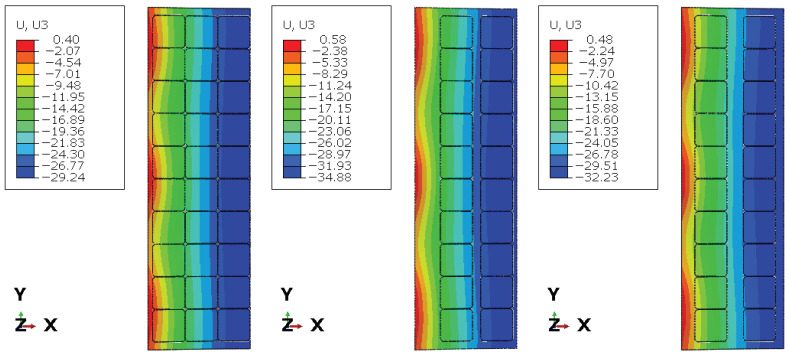
Displacements $u_{z}$ for Group I, II and III (left half).

**Figure 7:**
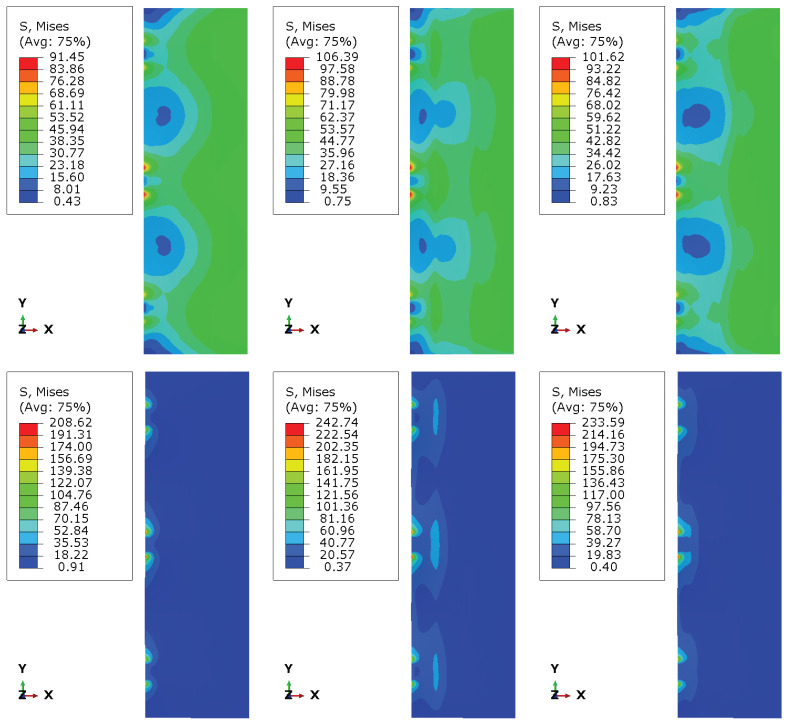
Mises stress $\sigma_{Mises}$ for Groups I, II and III (top and rear glass plies).

**Figure 8:**
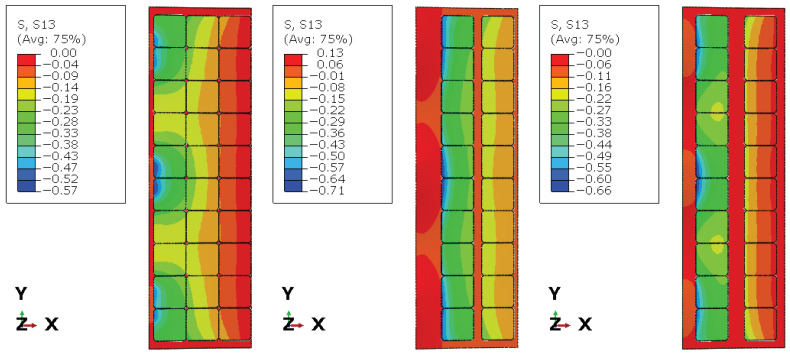
Shear stress $\sigma_{xz}$ for Groups I, II and III in the adhesive layer (POE).

Figure 6, Figure 7 and Figure 8 present the numerical distributions of out-of-plane displacements, equivalent von Mises stresses in the glass plies, and interlayer shear stresses in the adhesive layer for Groups I, II, and III, respectively.

The displacement contours $u_{z}$ shown in Figure 6 clearly illustrate the influence of photovoltaic cell layout on the global bending response. Group I, characterised by a dense and regular cell distribution, exhibits the smallest maximum deflection ($u_{z}\approx -29.24$ mm) and a relatively smooth displacement gradient through the panel width, indicating near-composite behaviour of the laminate. In contrast, Group II shows a pronounced increase in deflection ($u_{z}\approx -34.88$ mm), accompanied by stronger curvature of the displacement field, reflecting a significant loss of composite action due to extended regions without effective shear transfer. Group III exhibits an intermediate response ($u_{z}\approx -32.23$ mm), consistent with its more uniform but sparse cell arrangement.

The von Mises stress distributions in the glass plies, presented in Figure 7 for the top and rear glass plies, remain relatively smooth for all configurations and are dominated by bending stresses. Peak stress levels increase slightly from Group I to Group II (16%) and from Group I to Group III (11–12%), reflecting the higher curvature associated with reduced interlayer interaction. However, no strong stress concentrations are observed near the photovoltaic cells, indicating that the global bending response governs the stress state in the glass rather than local discontinuities in the interlayer.

**Figure 9:**
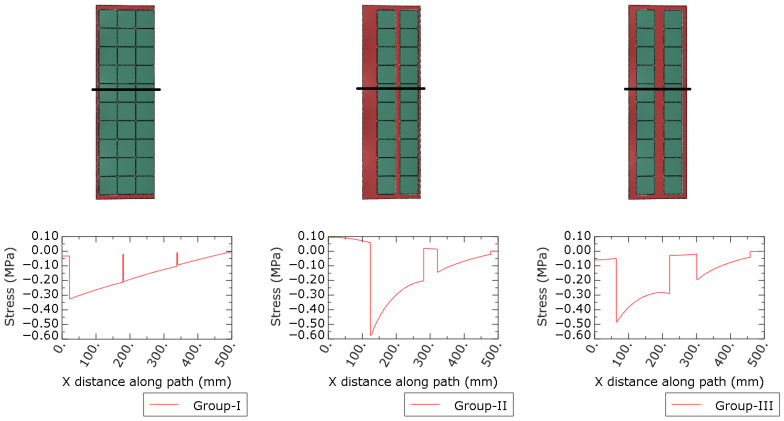
Distribution of the shear stress $\sigma_{xz}$ from Figure 8 along the marked black path for Groups I, II and III in the adhesive layer (POE).

Figure 8 and Figure 9 show the distribution of the shear stress component $\sigma_{xz}$ in the adhesive layer. For Group I, the shear stress field is relatively continuous along the panel length, with frequent stress transfer paths provided by the closely spaced photovoltaic cells acting as shear bridges. In contrast, Group II exhibits large regions with very low shear stress between the cells, indicating ineffective shear transfer over extended distances. Group III shows a more fragmented but still partially continuous shear stress pattern, consistent with its intermediate global stiffness. These results confirm that the spacing and distribution of photovoltaic cells directly control the effective shear transfer capacity of the interlayer.

The internal cell layout significantly influences the local stress distribution in the vicinity of silicon cells. In particular, increased inter-cell spacing leads to stress localization near cell edges and transitions between cell regions and gaps, which is consistent with the observed structural response and highlights the importance of explicitly modeling discrete cell geometry.

Overall, the stress and displacement fields consistently demonstrate that the experimentally observed differences in global stiffness are not caused by local stress concentrations, cracking, or material degradation. Instead, they arise from a structural loss of composite action governed by the spatial continuity of shear transfer in the interlayer and the resulting global kinematic constraints.

## 6. Alternative Modeling Approaches

Several alternative modeling strategies were considered and subsequently rejected, as they failed to reproduce the experimentally observed loss of stiffness in panels with low photovoltaic cell density or led to non-physical numerical behaviour.

Further reduction of the Young’s modulus of the adhesive in continuous solid models resulted primarily in non-physical softening in the thickness direction, without introducing a sufficient loss of interlayer shear transfer along the panel span. Even for significantly reduced stiffness values, the predicted deflections remained well below the experimental measurements, indicating that stiffness degradation alone is not the governing mechanism.

Hyperelastic material models were also assessed. However, under the considered loading regime the response remains fully reversible, and hyperelasticity does not introduce any additional mechanism for interaction loss between the glass plies. Consequently, such models yield results comparable to those obtained with linear elastic formulations.

Plasticity-based material descriptions were rejected because they inherently introduce irreversible deformations and path dependence. This behaviour is inconsistent with the experimentally observed symmetry of the response under load reversal, which indicates the absence of permanent deformation or damage in the adhesive layer.

Simplified shell-based or homogenized sandwich models were examined as reduced-order alternatives. A composite shell model using S4R elements (10 mm size) was constructed, consisting of three layers in the regions between the photovoltaic cells and five layers in the cell regions, Figure 1. For an isotropic adhesive representation, the predicted mid-span deflections were 30.14 mm for Group I, 30.57 mm for Group II and 30.44 mm for Group III, showing only marginal sensitivity to the cell distribution.

**Figure 10:**
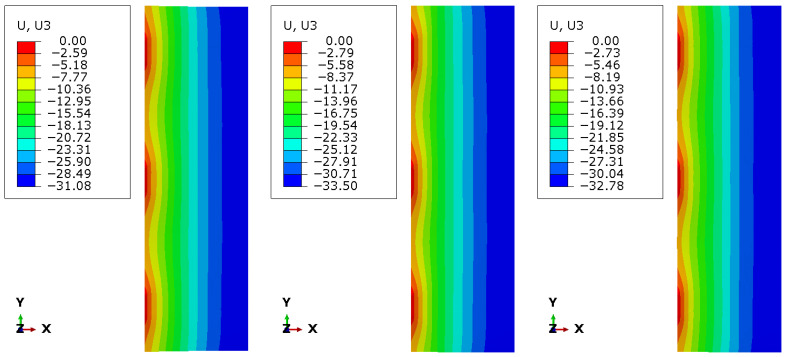
Deflection for the composite shell model for three Groups I, II and III (left half).

Introducing orthotropic material behaviour for the adhesive layers between the cells increased the deflections to 31.8 mm for Group I, 33.50 mm for Group II and 32.78 mm for Group III, Figure 10. Although this modification enhanced the differentiation between the configurations, the results remained below the experimental values. Further reduction of the shear modulus below G=1.8 MPa led to numerical convergence problems and the absence of stable solutions, indicating that the shell formulation is unable to accommodate very low interlayer shear stiffness in a physically consistent manner.

The obtained results demonstrate that shell-based or homogenized laminate models inherently enforce global kinematic constraints and suppress long-range shear-lag effects and they are unable to capture the pronounced loss of composite action observed in panels with sparse or uneven photovoltaic cell layouts.

## 7. Cycling Loading—Numerical Simulation

**Figure 11:**
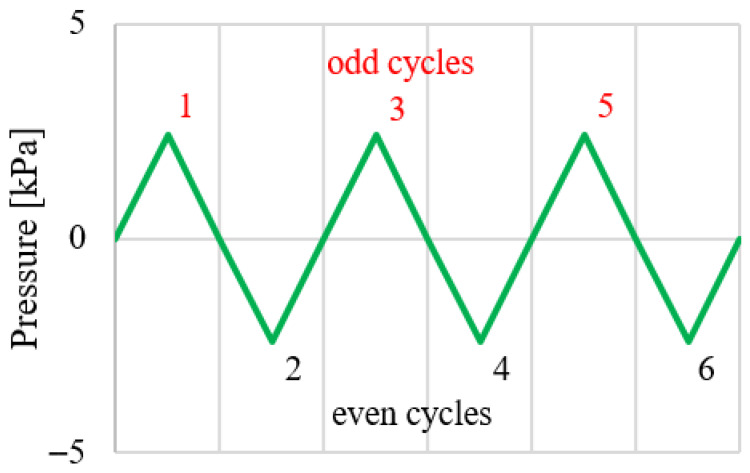
Loading in odd cycles (1, 3, 5) and even cycles (2, 4, 6).

**Figure 12:**
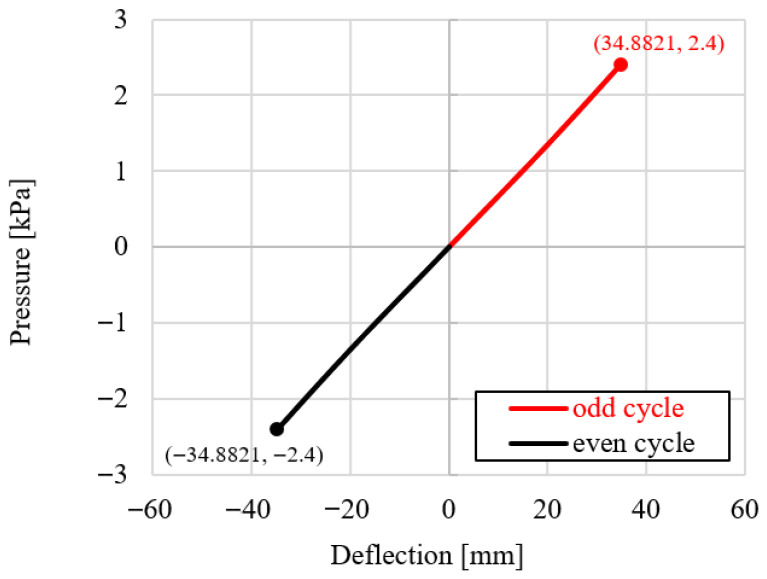
Reversible pressure–deflection behaviour of Group II under cyclic loading.

For Group II, an additional numerical simulation was performed in which the applied load was reversed, inducing bending in the opposite directions, Figure 11. A total of six loading–unloading cycles of the photovoltaic panels under sign-changing uniform pressure (with limit values of ±2.4 kPa) were applied. The corresponding pressure–deflection response is presented in Figure 12. The load–deflection curves obtained for the odd and even loading cycles are linear, symmetric with respect to the origin, and exhibit identical slopes. This indicates that the effective bending stiffness of the panel is the same in both loading directions, confirming a fully reversible response and the absence of stiffness degradation or directional dependence under cyclic loading. The same effect was observed in experimental tests for all panel groups subjected to a few-hour loading period [24].

## 8. Conclusions and Future Work

This study, as a continuation of the laboratory experiments described in [24], presents a numerical approach to investigate the mechanical response of bifacial glass–glass photovoltaic panels subjected to snow-type loading, with particular emphasis on the influence of silicon cell layout and the adopted modeling strategy. Good agreement between the obtained numerical results and the experimental findings was achieved.

Based on the laboratory experiments and numerical analyses performed, the following conclusions can be drawn:

- The global out-of-plane deformation of the panel is affected by the spacing of the silicon cells, while the boundary conditions and overall laminate stiffness govern the bending response.
- An important role is played by the boundary conditions and the modeling approach, which should allow for mutual slip of the cover glass plies in the regions outside the silicon cells.
- The internal cell layout has a pronounced influence on the stress state in the silicon cell layer and the adhesive layer, with increased inter-cell spacing leading to significant stress localization near cell edges and cell-gap transitions.
- The studied bifacial glass–glass photovoltaic panels, despite undergoing large reverse displacements—as measured by a sag-to-span ratio of approximately 1/30—exhibited practically no loss of electrical efficiency.
- Three-dimensional solid models, which explicitly represent the layered structure and discrete cell geometry, are essential for the reliable assessment of stress levels governing crack initiation and mechanical durability.

From a design perspective, the obtained results demonstrate that the silicon cell layout should be treated as a parameter influencing not only electrical performance but also mechanical behaviour of bifacial photovoltaic panels. Increased spacing between photovoltaic cells reduces interlayer shear transfer and leads to a decrease in effective stiffness. This is particularly relevant for BIPV and semi-transparent panels, where large cell gaps are introduced, and may affect serviceability criteria such as deflection limits under snow loading.

We experimentally determined that a few-hour loading period induced an elastic response of the panels. However, after removing the same load following 120 h of sustained loading, some residual displacements (4.6% of the maximum total displacement) were observed. The observed residual displacements indicate the presence of time-dependent effects, such as creep or relaxation. These effects may result from the viscoelastic behaviour of the adhesive interlayer (POE/EVA), particularly under sustained loading and varying temperature conditions, and may influence interlayer shear transfer and long-term stiffness. Further research is planned to investigate and model the long-term thermo-mechanical behaviour of the panels and to develop computational models accounting for temperature- and time-dependent phenomena.

## Figures and Tables

**Table 1:** Material parameters used in the numerical models.

Material	*E* [MPa]	ν [-]	Remarks	Models
Glass ply	73,000	0.24	Isotropic linear elastic	M1–M5
Photovoltaic cell (Si)	160,000	0.22	Isotropic linear elastic	M1–M5
Adhesive (under/above cells)	10	0.48	Nearly incompressible	M1–M5
Adhesive (around cells)	10	0.48	Nearly incompressible	M1
Adhesive (around cells)	3	0.48	Reduced stiffness	M2
Orthotropic adhesive	10	0.48	$G_{xz}=0.1$ MPa	M3
Frictionless contact (around cells)			between glass and adhesive	M4
Cohesive interface	$K_{n}=10$	–	$K_{s}=K_{t}=0.1$–0.5 MPa	M5

**Table 2:** Summary of modeling strategies.

Model	Interlayer Modeling	Shear Stiffness Assumption	Boundary Conditions	Physical Interpretation
M1	Continuous isotropic	Full	Joint for both plies	Full composite action
M2	Continuous isotropic (heterogeneous)	Reduced between cells	Joint for both plies	Partial stiffness reduction
M3	Continuous orthotropic	Reduced shear transfer	Joint for both plies	Shear-limited behaviour
M4	Discontinuous (contact)	No tangential transfer in gaps	Joint for both plies	No long-range shear transfer
M5	Discontinuous (cohesive)	Limited tangential transfer	Independent for each ply	Partial interaction with slip

**Table 3:** Comparison of maximum out-of-plane deflections obtained from 3D solid models with experimental results [24] for different silicon cell layout groups.

Group	Simulation [mm]	Experiment [mm]	Relative Error [%]
Group I	29.24	28.6	2.2
Group II	34.88	34.8	0.2
Group III	32.23	34.0	5.2

## Data Availability

The original contributions presented in this study are included in the article. Further inquiries can be directed to the corresponding authors.
